# Supplementary material for: Electrothermally controlled origami fabricated by 4D printing of continuous fiber-reinforced composites
Source: Nat Commun. 2024 Mar 14;15:2322. doi: 10.1038/s41467-024-46591-3 (PMC10940589; doi:10.1038/s41467-024-46591-3)
Supplement: Supplementary file 1 — Supplementary Information [file 41467_2024_46591_MOESM1_ESM.pdf]

Supplementary Information for

Electrothermally controlled origami fabricated by 4D printing of continuous fiber-reinforced composites

Yaohui Wang[1,4], Haitao Ye[2,3,4], Jian He[1], Qi Ge[2]*, Yi Xiong[1]*

[1]School of System Design and Intelligent Manufacturing, Southern University of Science and Technology, Shenzhen 518055, China.

[2]Department of Mechanical and Energy Engineering, Southern University of Science and Technology, Shenzhen 518055, China.

[3]Department of Mechanical Engineering, City University of Hong Kong, Kowloon, Hong Kong SAR, China.

[4]These authors contributed equally: Yaohui Wang, Haitao Ye.

*Corresponding author. Email: geq@sustech.edu.cn (Q.G.); xiongy3@sustech.edu.cn (Y.X.)

This PDF file includes:

Supplementary Text
Supplementary Figures 1 to 27
Supplementary Tables 1 to 4

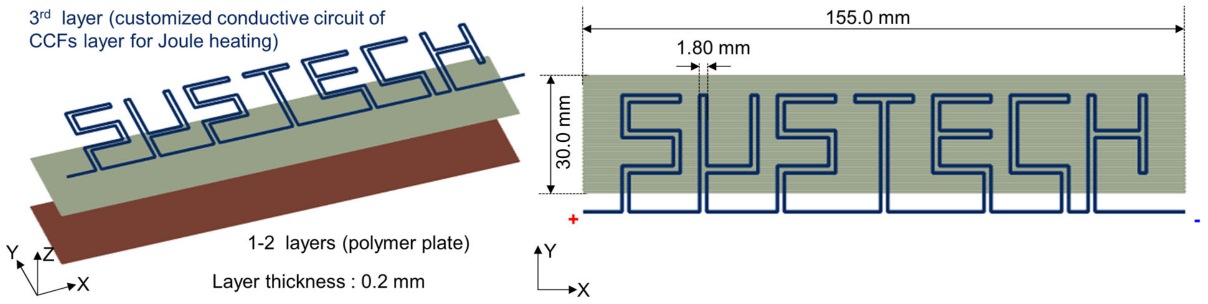

Supplementary Fig. 1. Construction and dimensions of the customized conductive circuit. 1–2 layers are printed by the left nozzle of the 3D printer (Anisoprint A4, Anisoprint, Russia), which with a diameter of 0.4 mm supplies thermoplastics (PLA here) only. The third layer of the CCFs layer is printed by the right nozzle with a diameter of 1 mm and fed CCFs impregnated with thermoplastics.

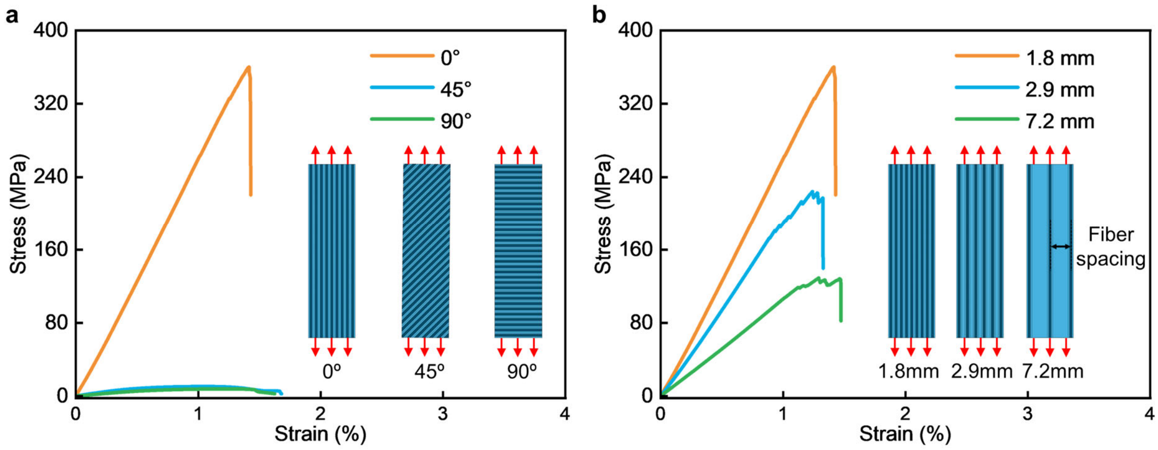

Supplementary Fig. 2. Tensile results of specimens with different fiber arrangement parameters. a Stress-strain curves of CCF-SMP specimen with different fiber angles. **b** Stress-strain curves of CCF-SMP specimen with different fiber spacing.

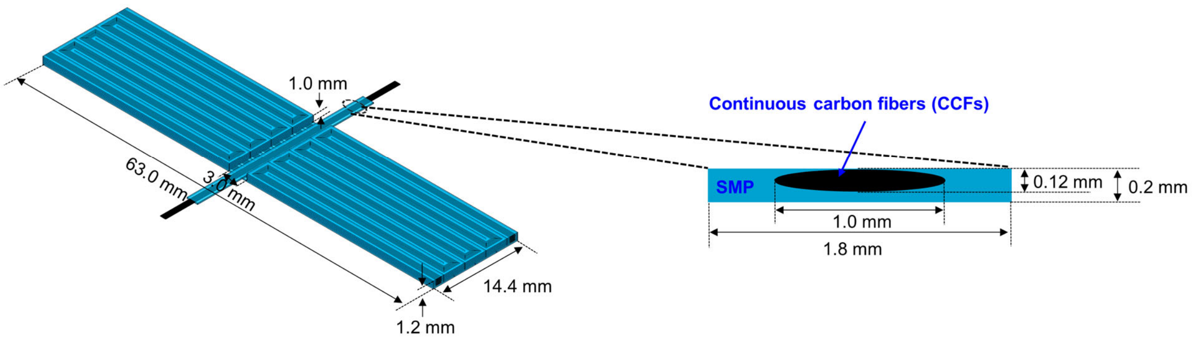

Supplementary Fig. 3. Construction and dimensions of the hinged strip.

Supplementary Notes

Supplementary Note 1. Calculation of temperature-time curve of continuous carbon fibers

In order to simulate and analyze the thermal distribution inside the hinge when the continuous carbon fibers (CCFs) are applied with different currents for triggering shape-shifting, the temperature-time curve of the CCFs with different currents is calculated numerically. Some necessary parameters related to CCFs are characterized.

When voltage is applied to both ends of CCFs, heat is generated due to the flow of current, accompanied by continuous heat dissipation, and the temperature of CCFs gradually stabilizes after a period. According to the heat balance principle (i.e., thermal equilibrium), the heat Q_1 generated by Joule heating of CCFs is equal to the sum of the heat consumption Q_2 and convection-based heat dissipation Q_3 :

$$Q_1 = Q_2 + Q_3. \tag{1}$$

The increment of each item is $dQ_1 = I^2R(T)dt$, $dQ_2 = mcdT$, and $dQ_3 = K_{\mathrm{F}}A(T - T_0)dt$, which also follows the heat balance principle and is given by

$$I^2R(T)dt = mcdT + K_{\mathrm{F}}A(T - T_0)dt, \tag{2}$$

where I is the current, $R(T)$ is the resistance of CCFs, which is a function of the temperature T of CCFs, m, c, K_{F} , and A are respectively the mass, specific heat capacity, overall heat transfer coefficient, and heat dissipation area of CCFs, and T_0 is the ambient temperature. The resistance of CCFs can be expressed as

$$R(T) = \rho_0(1 + \alpha_{\mathrm{F}}(T - T_0))\frac{L}{S}, \tag{3}$$

where ρ_0 is the resistivity of CCFs at T_0, and α_{F}, L, and S are respectively the temperature coefficient of resistance, length, and cross-sectional area of CCFs. Then, Equation (2) can be expressed as follows

$$I^2\rho_0(1+\alpha_{\mathrm{F}}(T-T_0))\frac{L}{S}dt = mcdT + K_{\mathrm{F}}A(T-T_0)dt \tag{4}$$

$$I^2\rho_0(1+\alpha_{\mathrm{F}}(T_{\mathrm{s}}-T_0+T-T_{\mathrm{s}}))\frac{L}{S}dt = mcdT + K_{\mathrm{F}}A(T_{\mathrm{s}}-T_0+T-T_{\mathrm{s}})dt \tag{5}$$

$$I^2\rho_0(1+\alpha_{\mathrm{F}}(T_{\mathrm{s}}-T_0))\frac{L}{S}dt + I^2\rho_0\alpha_{\mathrm{F}}(T-T_{\mathrm{s}})\frac{L}{S}dt = mcdT + K_{\mathrm{F}}A(T_{\mathrm{s}}-T_0)dt + K_{\mathrm{F}}A(T-T_{\mathrm{s}})dt \tag{6}$$

For the stable state, the temperature reaches a stable value T_{s}, $dT = 0$, then $dQ_2 = 0$, $dQ_1 = dQ_3$, Equation (4) can be expressed as

$$I^2\rho_0(1+\alpha_{\mathrm{F}}(T_{\mathrm{s}}-T_0))\frac{L}{S} = K_{\mathrm{F}}A(T_{\mathrm{s}}-T_0). \tag{7}$$

Then the T_{s} can be calculated as

$$T_{\mathrm{s}} = T_0 + \frac{I^2\rho_0 L}{K_{\mathrm{F}}AS - I^2\rho_0\alpha_{\mathrm{F}}L}. \tag{8}$$

Combine Equations (6) and (7), we can have:

$$I^2\rho_0\alpha_{\mathrm{F}}(T-T_{\mathrm{s}})\frac{L}{S}dt = mcdT + K_{\mathrm{F}}A(T-T_{\mathrm{s}})dt \tag{9}$$

$$\frac{(I^2\rho_0\alpha_{\mathrm{F}}L - K_{\mathrm{F}}AS)}{mcS}dt = \frac{1}{T-T_{\mathrm{s}}}dT \tag{10}$$

Combine Equations (8) and (10), we can have:

$$-\frac{I^2\rho_0 L}{mcS(T_{\mathrm{s}}-T_0)}dt = \frac{1}{T_{\mathrm{s}}-T}d(T_{\mathrm{s}}-T). \tag{11}$$

After integration, we can have:

$$\int_0^t -\frac{I^2 \rho_0 L}{mcS(T_s - T_0)} dt = \int_{T_0}^{T} \frac{1}{T_s - T} d(T_s - T) \tag{12}$$

$$-\frac{I^2 \rho_0 L}{mcS(T_s - T_0)} t = \ln \frac{T_s - T}{T_s - T_0} \tag{13}$$

$$\exp(-\frac{I^2 \rho_0 L}{mcS(T_s - T_0)} t) = \frac{T_s - T}{T_s - T_0} \tag{14}$$

Let $\tau_F = mcS(T_s - T_0) / I^2 \rho_0 L$, which is the thermal time constant of CCFs. Then, the temperature T is a function of time t, which can be expressed as

$$T(t) = T_0 + (T_s - T_0)(1 - \exp(-t / \tau_F)), \tag{15}$$

where T_s and τ_F are functions of I, ρ_0, α_F, and K_F, which follows as

$$T_s(I, \rho_0, \alpha_F, K_F) = T_0 + \frac{I^2 \rho_0 L}{K_F AS - I^2 \rho_0 \alpha_F L} \tag{15a}$$

$$\tau_F(I, \rho_0, \alpha_F, K_F) = \frac{mcS}{K_F AS - I^2 \rho_0 \alpha_F L} \tag{15b}$$

Combine Equations (2) and (15), we can calculate the body heat flux $\dot{\Phi}_{JH}(t)$ of CCFs given by

$$\dot{\Phi}_{JH}(t) = \frac{I^2 R(T)}{V} = \frac{I^2 \rho_0}{S^2}(1 + \alpha_F (T_s - T_0)(1 - \exp(-t / \tau_F))), \tag{16}$$

where V is the volume of CCFs.

In this work, the ambient temperature T_0 of 25°C, and the specific heat capacity c of 1950 J/(Kg·K). Other parameters related to CCFs are measured as follows: the length L, cross-sectional area S, and heat dissipation area A are 0.1 m, 9.6×10^{-8} m^2, and 1.1×10^{-4} m^2, respectively, the resistivity ρ_0 of 2.97×10^{-5} Ω·m (measured by Supplementary Fig. 3a), the temperature coefficient of resistance α_F of -0.0499 %/°C (measured by Supplementary Fig. 3b), and the overall heat transfer

coefficient K_F obtained based on Equations (6) and (15) and experimental fitting (Supplementary Fig. 3c), is 120.8 W/(K·m^2). The body heat flux $\dot{\Phi}_{JH}$ of CCFs with different applied currents is calculated in Supplementary Fig. 4.

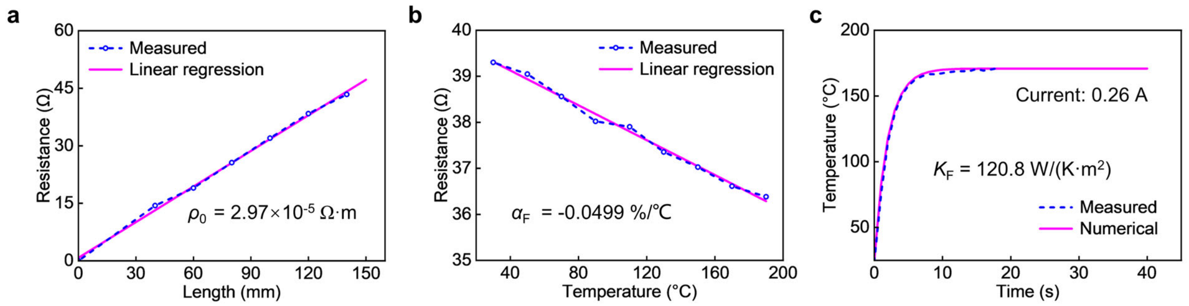

Supplementary Fig. 4. Measurement of necessary parameters of CCF. **a** The resistivity of CCF. Both ends of the CCFs burn off the resin and are clamped by a multimeter (FLUKE 15B+) to measure the resistance. **b** Temperature coefficient of resistance of CCFs. Both ends of the CCFs burn off the resin and are clamped by a power supply (GPD-4303S, GWinstek, China) to measure the resistance, and a thermal imager (Co. MAG 32, Magnity Technologies, China) is used to record the temperature. **c** Overall heat transfer coefficient of CCFs. Both ends of the CCFs burn off the resin and are clamped by a power supply (GPD-4303S, GWinstek, China) to supply current (0.26A), and a thermal imager (Co. MAG 32, Magnity Technologies, China) is used to record the temperature.

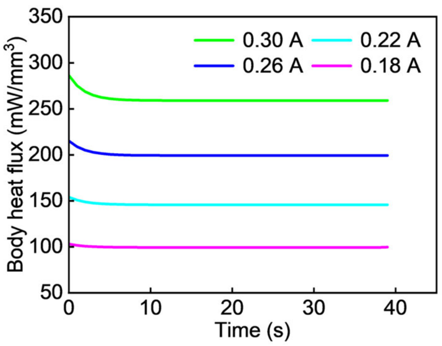

Supplementary Fig. 5. The body heat flux of CCFs with different applied currents.

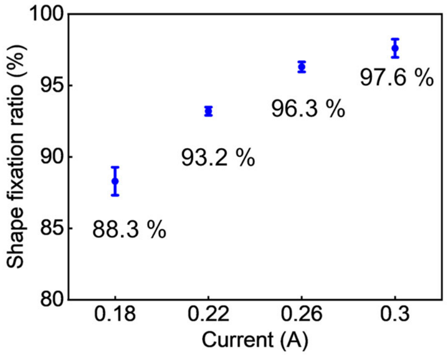

Supplementary Fig. 6. Shape fixation ratio of CCF-SMP hinged strip with different applied currents. Three tests are conducted on samples for each data point, and the error bars present the standard deviation of the three repeated data.

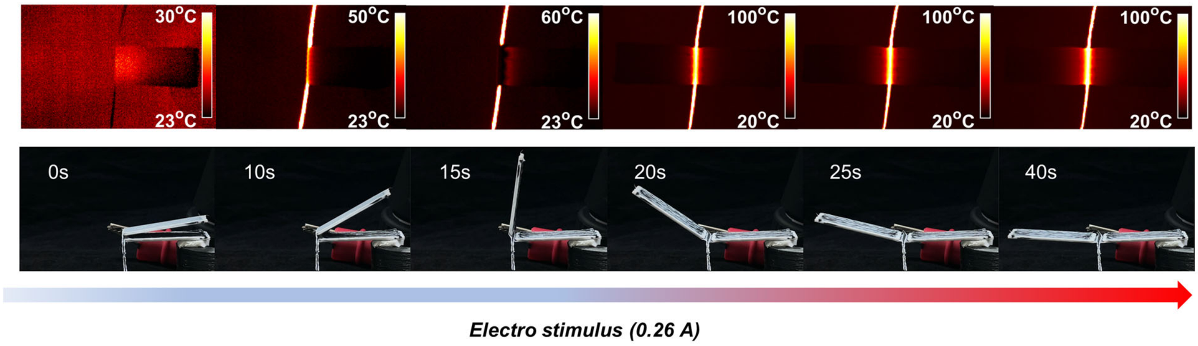

Supplementary Fig. 7. Electro-induced shape memory behavior of CCF-SMP hinged strip is recorded by thermal imager (Co. MAG 32, Magnity Technologies, China) and digital camera (Nikon, Z7II). The temperature data is processed by Matlab software and the video is processed by Adobe After Effects 2022 software.

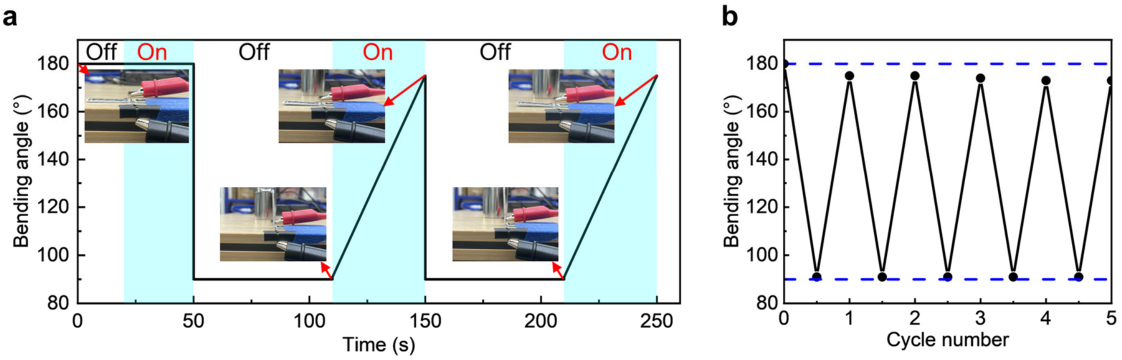

Supplementary Fig. 8. Shape transformation and recovery of a CCF-SMP hinged strip. a Shape transformation and recovery of a CCF-SMP hinged strip under the electrical stimulus is 0.26 A. **b** Repeatability of the CCF-SMP hinged strip under the electrical stimulus is 0.26 A.

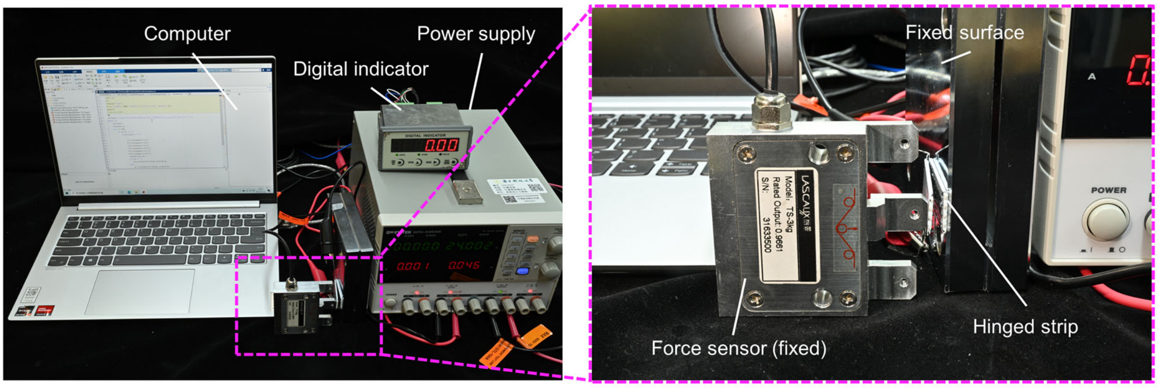

Supplementary Fig. 9. Experimental setup for the shape recovery force measurement. Shape recovery force data of the hinged strip is collected by a force sensor (LS30, Lijing, China), whose measuring range is -30 to 30 N.

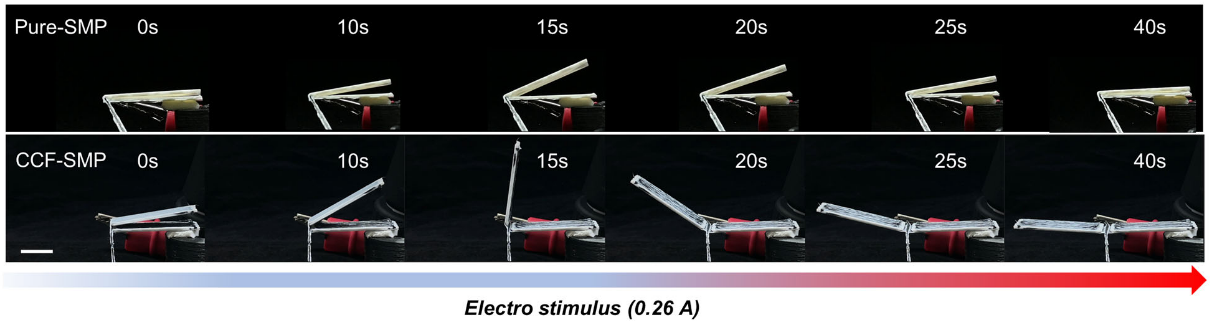

Supplementary Fig. 10. Influence of shape recovery force on the deployment of the PCEO. Snapshots of electro-induced shape memory behavior of pure-SMP and CCF-SMP hinged strip structure. Scale bar, 10 mm.

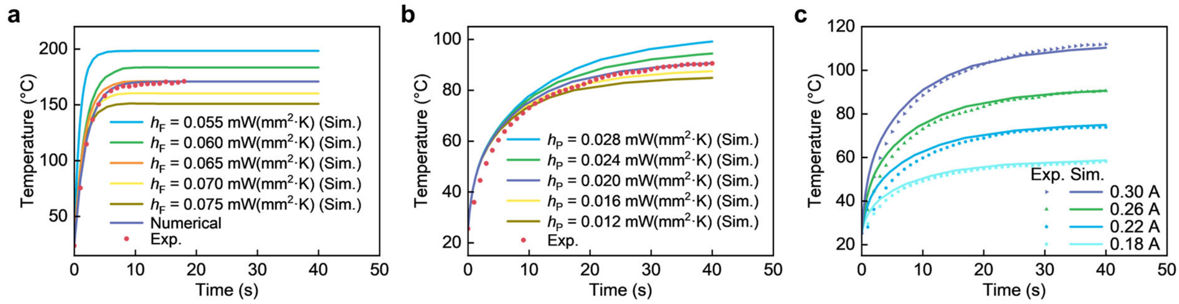

Supplementary Fig. 11. The determination of the convection coefficient. It should be noted that the convection coefficient between air and different materials varies. For convection between air and fibers, h is denoted as h_F. For convection between air and polymers, h is denoted as h_P. **a** The determination of the convection coefficient of fibers by simulating the heating of CCFs with an applied current of 0.26 A. **b** The determination of the convection coefficient of polymers by simulating the heating of hinges with an applied current of 0.26 A. **c** The validation of their effectiveness. It should be noted that the heat transfer between the structure and the surrounding environment is simplified as a heat convection problem. The convection coefficient of fibers is determined to be 0.065 mW/(mm2·K) and that of polymers is 0.020 mW/(mm2·K).

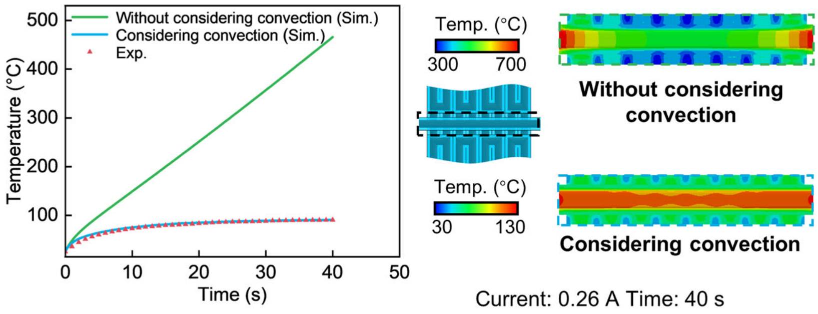

Supplementary Fig. 12. Comparison of the hinge heating and thermal distribution with and without considering convection under 0.26 A Joule heating.

Supplementary Note 2. Multibranch thermoviscoelastic model of continuous carbon fiber-reinforced shape memory polymer.

To describe the thermomechanical response of CCF-SMP, we establish a multi-branch viscoelastic model[1, 2], in which parallel arranged two equilibrium elastic branches and multiple thermoviscoelastic nonequilibrium branches (as shown in Fig. 3b). $E_{\mathrm{eq\_P}}$ and $E_{\mathrm{eq\_F}}$ are the elastic modulus of the equilibrium branch of polymers and fibers. Maxwell elements are used in the nonequilibrium branches to represent the stress relaxation behavior of the material, E_i and τ_i are the elastic modulus and the temperature–dependent relaxation time of the *i*th nonequilibrium branch, respectively.

Since these relaxation times depend on temperature, CCF-SMP's response time also varies depending on temperature. By following the time-temperature superposition principle (TTSP), τ_i can be determined by using the relaxation time τ_i^r at the reference temperature:

$$\tau_i(T) = \alpha^{shift}(T)\tau_i^r, \tag{17}$$

where $\alpha^{shift}(T)$ is the time-temperature superposition shift factor. Shift factor can be calculated by using the Williams-Landel-Ferry (WLF) equation[3]:

$$\log_{10}\left[\alpha^{shift}(T)\right] = -\frac{C_1(T-T_r)}{C_2+(T-T_r)}, \tag{18}$$

where C_1 and C_2 are material parameters, T_r is the reference temperature. To obtain $\alpha^{shift}(T)$, as well as C_1 and C_2, stress relaxation tests at varied temperatures are conducted and then the fitted result of the time-temperature superposition shift factor is shown in Supplementary Fig. 9.

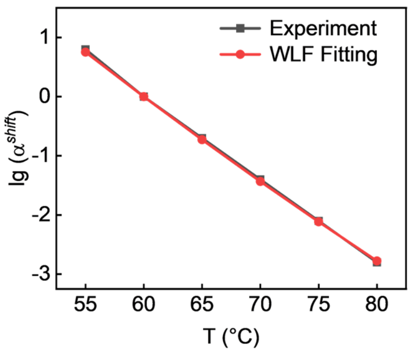

Supplementary Fig. 13. Shift factor α^{shift} vs. temperature.

For the multi-branch model, the temperature-dependent storage modulus $E_s(T)$, loss modulus $E_l(T)$ and $\tan\delta$ can be respectively represented as[1]

$$E_s(T) = E_{\mathrm{eq\_F}} + E_{\mathrm{eq\_P}} + \sum_{i=1}^{n} \frac{E_i \omega_{\mathrm{t}}^2 \left[\tau_i(T)\right]^2}{1+\omega_{\mathrm{t}}^2 \left[\tau_i(T)\right]^2} \tag{19}$$

$$E_l(T) = \sum_{i=1}^{n} \frac{E_i \omega_{\mathrm{t}} \tau_i(T)}{1+\omega_{\mathrm{t}}^2 \left[\tau_i(T)\right]^2} \tag{20}$$

$$\tan\delta = \frac{E_l(T)}{E_s(T)} \tag{21}$$

where ω_{t} is the test frequency. By fitting the storage modulus and $\tan\delta$ curve of the DMA test results (Supplementary Fig. 10), the elastic modulus E_i and relaxation time τ_i^r of each branch can be determined.

In this work, we use a reference temperature T_r of 60 °C. The elastic modulus E_{eq} ($E_{\mathrm{eq\_P}} + E_{\mathrm{eq\_F}}$) in the equilibrium branch is 633.73 MPa, which is chosen as the storage modulus at 90 °C. The values of C_1 and C_2 are respectively 44.9 and 303. The values of E_i and τ_i^r for the multi-branch model, are listed in Supplementary Table 1.

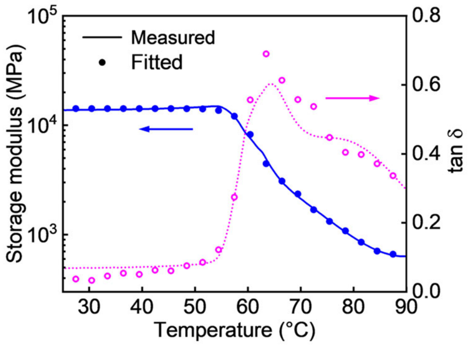

Supplementary Fig. 14. Fitting the storage modulus and tan δ between DMA test results and multi-branch thermoviscoelastic model.

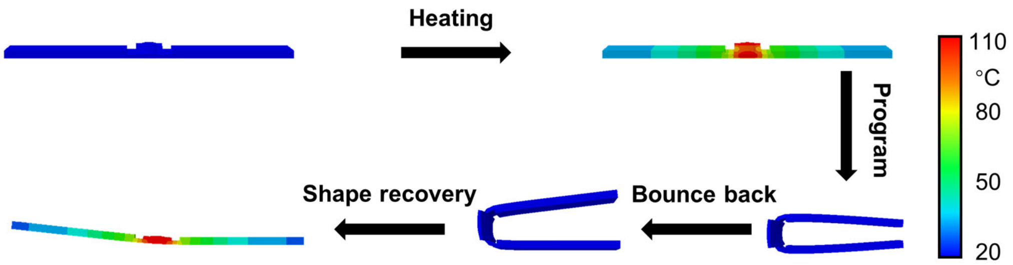

Supplementary Fig. 15. Simulation steps of the free recovery process of CFF-SMP hinge.

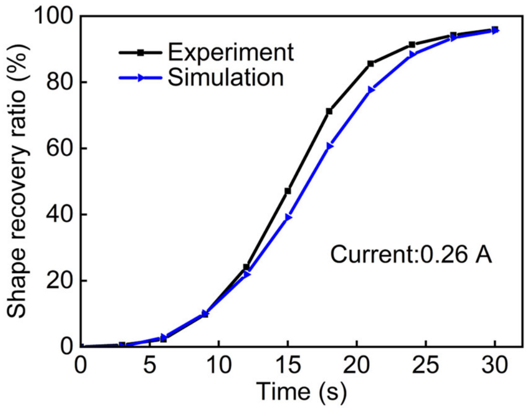

Supplementary Fig. 16. The shape recovery ratio of the shape recovery process of CCF-SMP hinge vs. time from experiment and simulation under 0.26 A Joule heating.

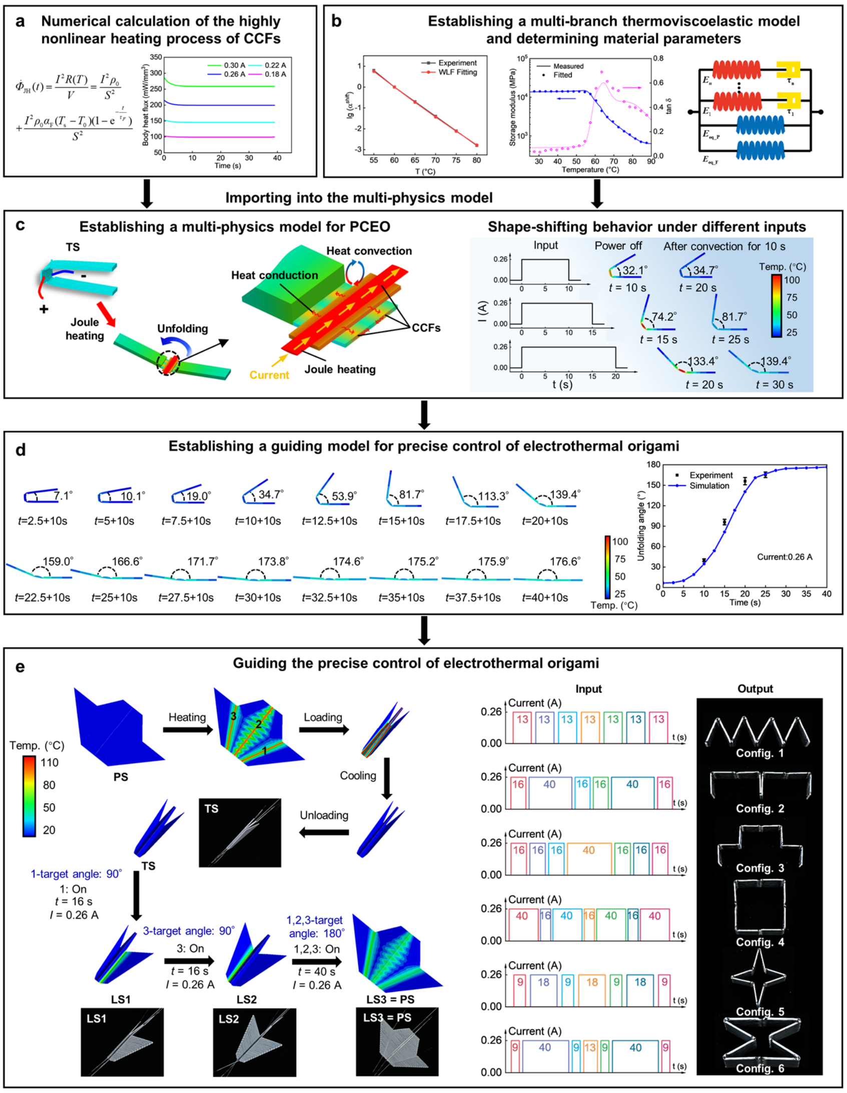

Supplementary Fig. 17. Establishment and guidance of the multi-physics model for precisely controlled electrothermal origami. a Numerical calculation of the highly nonlinear heating process of CCFs. **b** Establishment of the multi-branch thermoviscoelastic model for shape memory composites and the determination of the material parameters. **c** Import of the body heat flux of numerical calculation of CCFs and the material model, establishment of the multi-physics model, and simulation of the shape-shifting behaviors of electrothermal origami with different inputs. **d** Simulation results of the locked shape of the electrothermal origami with a heating time from 0 s to 40 s with a time interval of 2.5 s, and the establishment of the guiding model for the heating time corresponding to the locking angle. **e** Precise control of the airplane-shaped origami with complex deploying routines and the strip origami with reconfigurability guided by the guiding model.

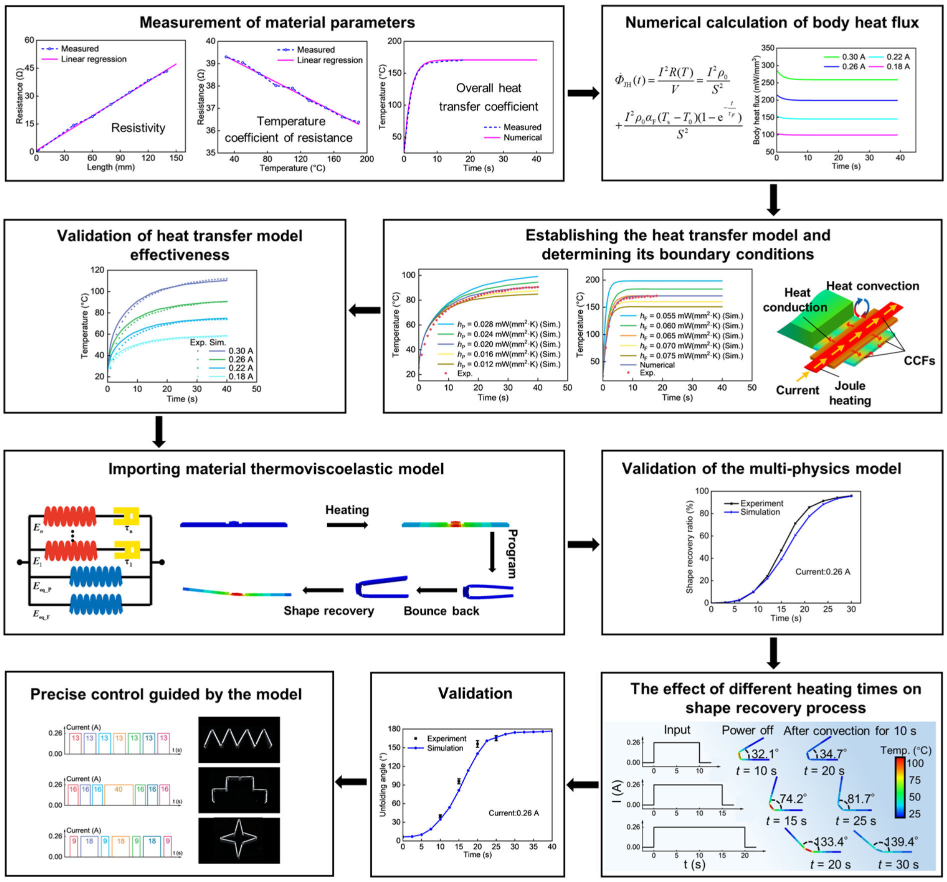

Supplementary Fig. 18. Flowchart of multi-physics simulation for precisely controlled electrothermal origami. The process includes nine steps: i) measuring the necessary parameters of CCFs for numerical calculation of its heating curve; ii) calculating the body heat flux of CCFs based on their heating curves under different currents; iii) importing the body heat flux of CCFs into the finite element model established for the heat transfer simulation of the electrothermal origami, and determining the boundary conditions (convection coefficients) of the model; iv) validating the effectiveness of the heat transfer model by comparing the hinge heating results of simulation and experiment under different currents; v) importing the multi-branch thermoviscoelastic model for shape memory composites into the simulation model, and creating the shape memory composites programming process; vi) validating the multi-physics model by comparing the shape recovery results of simulation and experiment; vii) simulating the shape-shifting behavior of the electrothermal origami under different heating times; viii) generating the guiding model for the heating time corresponding to the locking angle through simulation, and validating its effectiveness by conducting experiments; ix) guiding the precise control of the electrothermal origami by validated model.

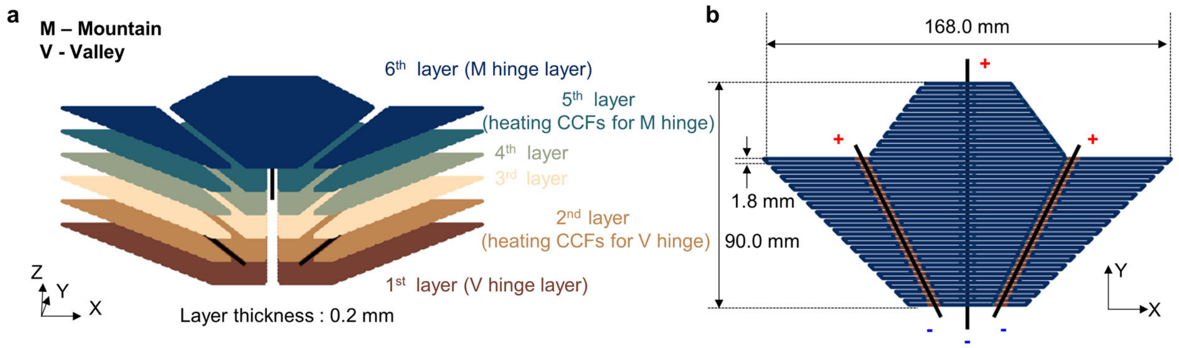

Supplementary Fig. 19. Construction and dimensions of the airplane-shaped origami structure. a Oblique view of fiber path of the airplane-shaped origami structure. There are a total of 6 layers, the first layer is the valley hinge layer, the last layer is the mountain hinge layer, and the second and fifth layers are CCFs layers, heating for the valley and mountain hinge layer, respectively. **b** Vertical view of fiber path and dimension of the airplane-shaped origami structure.

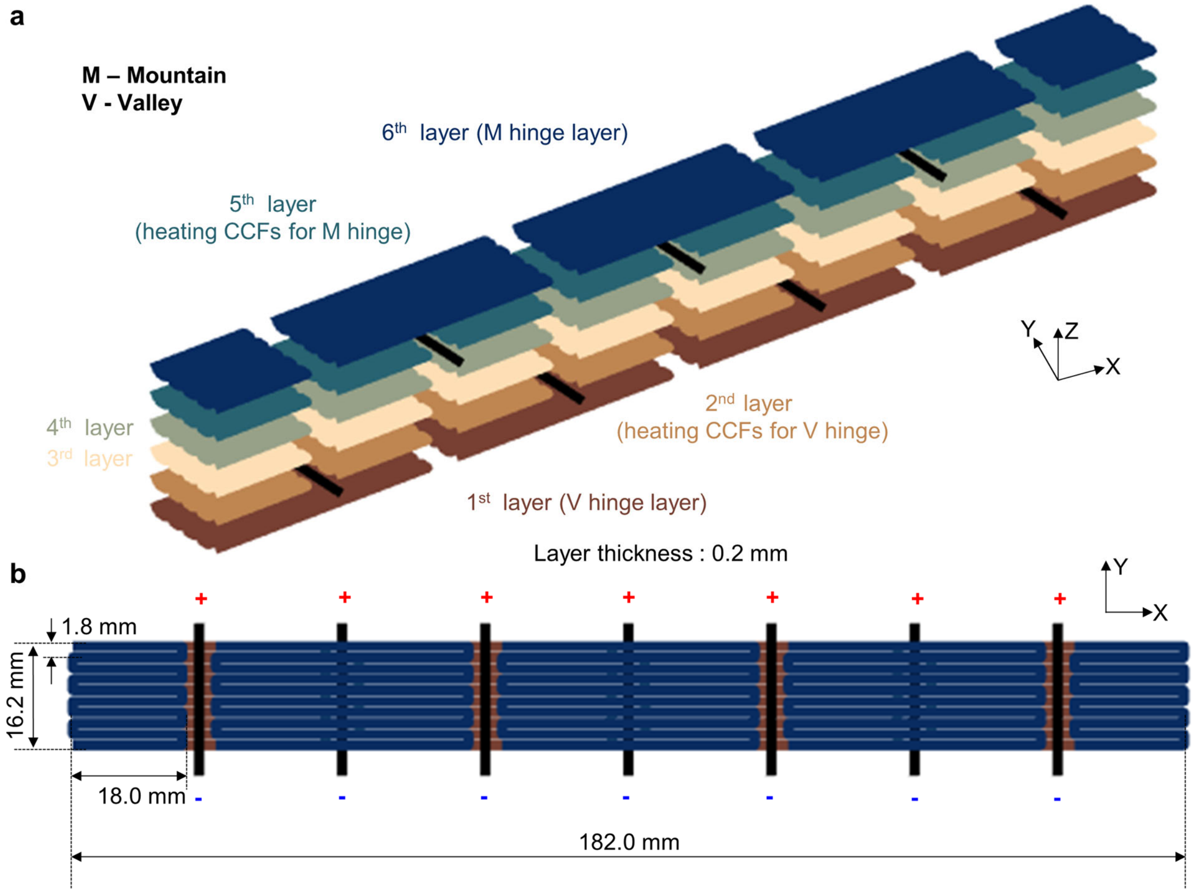

Supplementary Fig. 20. Construction and dimensions of the reconfigurable hinged strip structure. a Oblique view of the fiber path of the reconfigurable hinged strip structure. The first layer and the last layer are respectively the valley and mountain hinge layer, and the second and fifth layers are CCFs layers, heating for the valley and mountain hinge layer, respectively. **b** Vertical view of fiber path and dimension of the reconfigurable hinged strip structure.

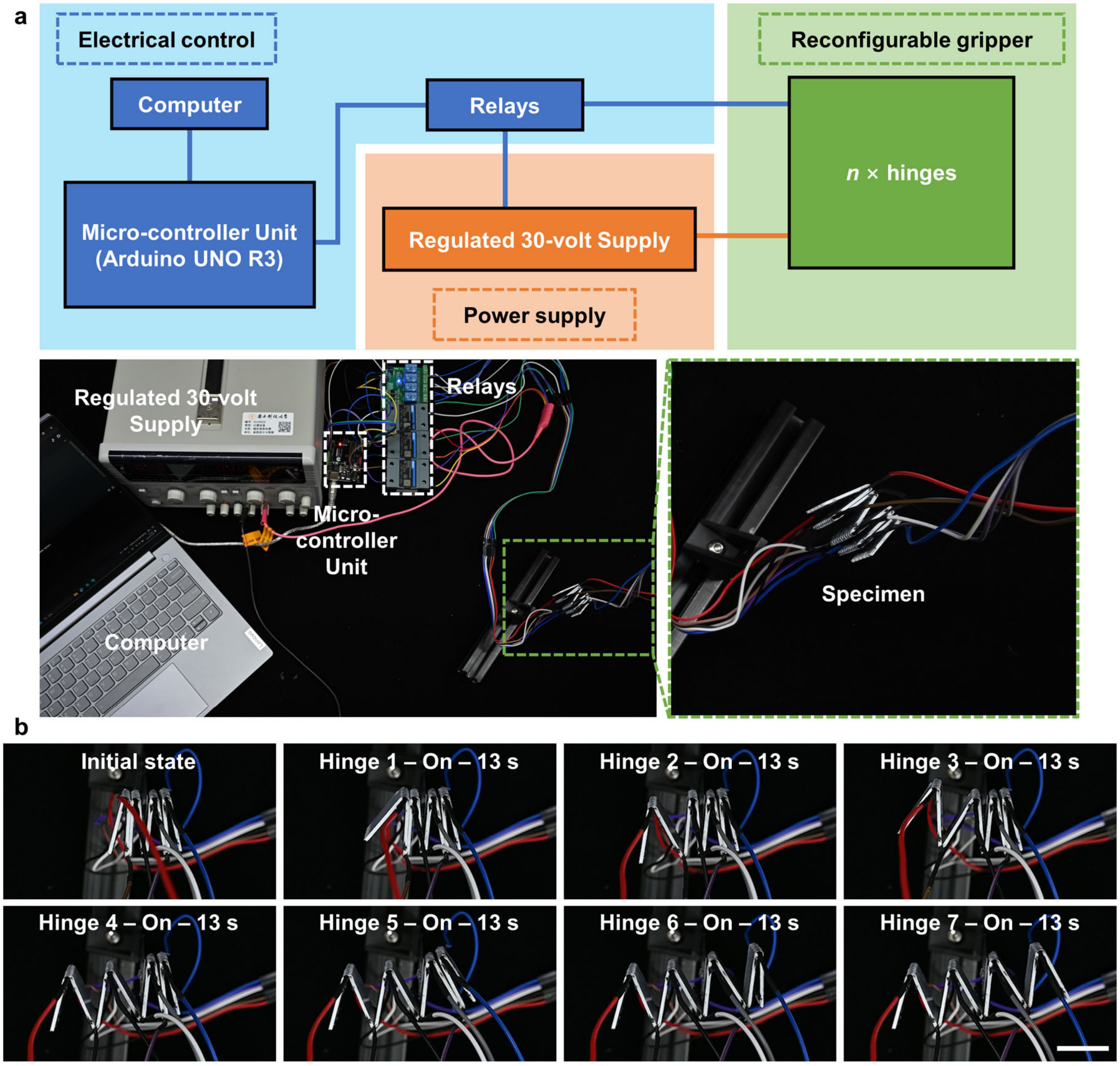

Supplementary Fig. 21. An automatic control method of the reconfigurable PCEO. a Control system of the reconfigurable PCEO. The control system consists of two parts: the power supply and control systems. The voltage was provided by a regulated a regulated 24-volt supply (GPD-4303S, GWinstek, China). The switches of the hinges are controlled by the signal from the microcontroller (Arduino UNO R3, DFROBOT, China) via relays (TOUGLESY, CHNT, China). **b** Deployment process of PCEO automatically realized by the self-built control system, Scale bar, 20 mm. Though the control method is out of the scope of this work, we demonstrate the feasibility of realizing automatic control of the reconfigurable PCEO by using this self-built control system. In this work, we chose a manual control method based on removing the alligator clip and manually reconnecting it with other wires, as presented in Supplementary Movie 4.

Supplementary Note 3. The internal geometric relationship of Miura-origami unit cell

A Miura unit cell is shown in Fig. 5b. Its geometry can be parameterized by a parallelogram with sides a and b and acute angle φ, and the deployment angle $\omega \in [0, \pi]$ between the side a and xy plane. The outer dimensions are then given by

$$H = a \cdot \sin\omega \tag{22}$$

$$S = 2b \cdot \sqrt{\frac{\cos^2\omega - \cos^2\varphi}{\cos^2\omega}} \tag{23}$$

$$L = 2a \cdot \cos\omega \tag{24}$$

and

$$V = b \cdot \frac{\cos\varphi}{\cos\omega}. \tag{25}$$

When designing Miura-origami metamaterials, the mechanical properties are often characterized in a partly folded state[4]. Four a selected set of outer dimensions *H, S, L,* and *V*, the crease pattern parameters can then inverse design. An additional useful relationship is

$$\cos\varphi = \cos\omega \cos\xi. \tag{26}$$

With $\varphi \in [0, \pi/2]$ and $\xi \in [0, \pi/2]$.

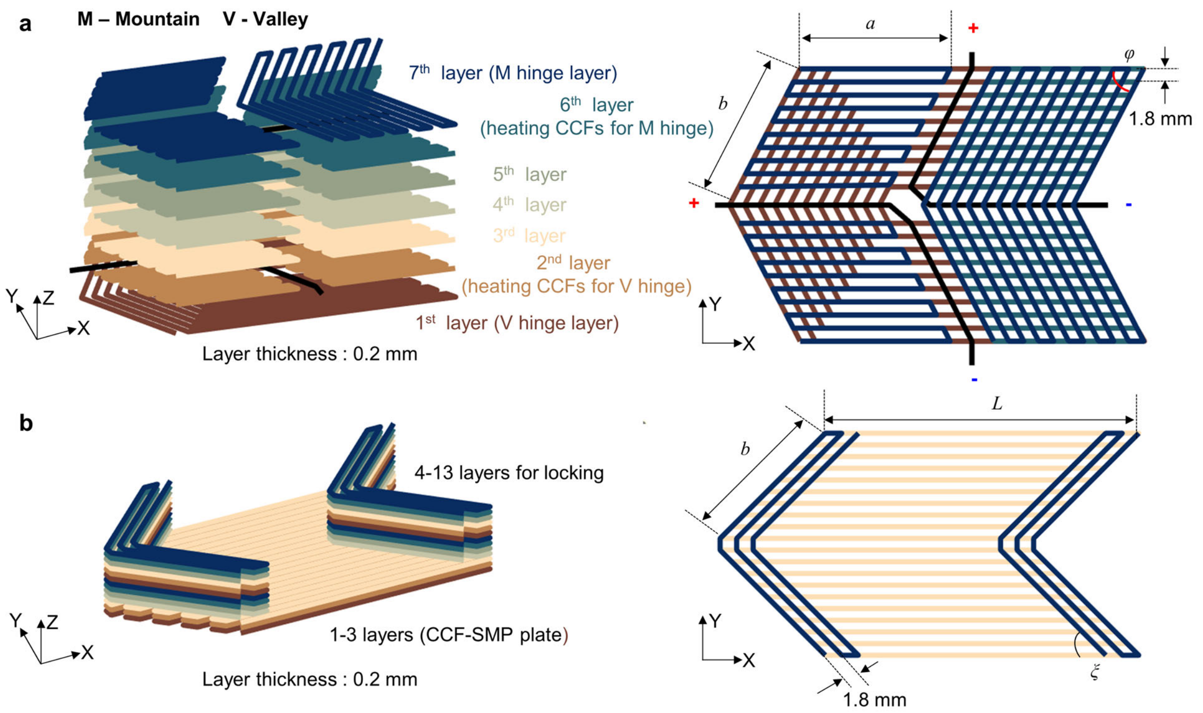

Supplementary Fig. 22. Construction and dimensions of the Miura-origami unit upper panel and limit bottom panel. a Oblique and vertical view of the fiber path of the Miura-origami unit upper panel. There are a total of 7 layers, the first layer and the last layer are respectively the valley and mountain hinge layer, and the second and sixth layers are CCFs layers, heating for the valley and mountain hinge layer, respectively. **b** Oblique and vertical view of fiber path of the limit bottom panel, where 1-3 layers for CCF-SMP plate and 4-13 layers for locking.

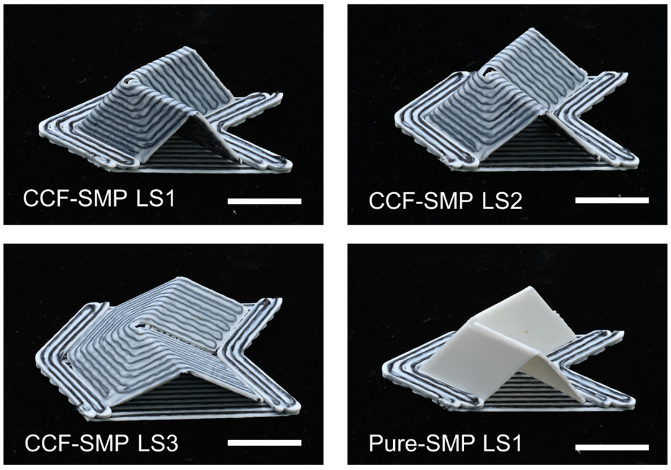

Supplementary Fig. 23. Snapshots for different three-dimensional deployment structures with a stopper bottom plate. Scale bars, 20 mm.

Supplementary Note 4. The inverse design of the geometry of Miura-origami unit for combinatory digital mechanical metamaterials.

In this work, the unit cell limit bottom plate of the combinatory digital mechanical metamaterial is designed as follows: ξ = 45°, L = 31.7 mm, and S = 27.7 mm. So, for a selected deployment angle ω, the crease pattern parameters a, b, and acute φ can be back-calculated as follows

$$a = f^{-1}(\omega) = \frac{L}{2 \cdot \cos\omega} = \frac{15.85}{\cos\omega} \tag{27}$$

$$b = \frac{S}{2 \cdot \sin\xi} = 19.6 \text{ mm} \tag{28}$$

$$\varphi = g^{-1}(\omega) = \cos^{-1}(\cos\omega\cos\xi) = \cos^{-1}(0.707\cos\omega) \tag{29}$$

and then

$$H = w^{-1}(\omega) = a \cdot \sin\omega = 15.85\tan\omega. \tag{30}$$

The inverse design of parameters a, φ, and H with a given ω is shown in Supplementary Fig. 17.

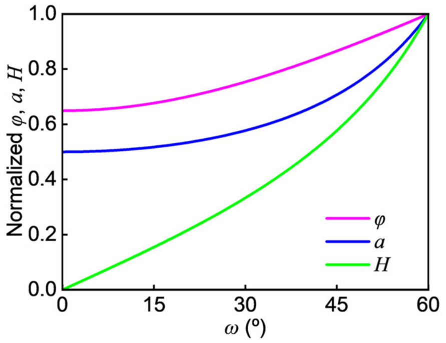

Supplementary Fig. 24. The inverse design of the geometry of the Miura-origami unit for combinatory digital mechanical metamaterials with a given ω.

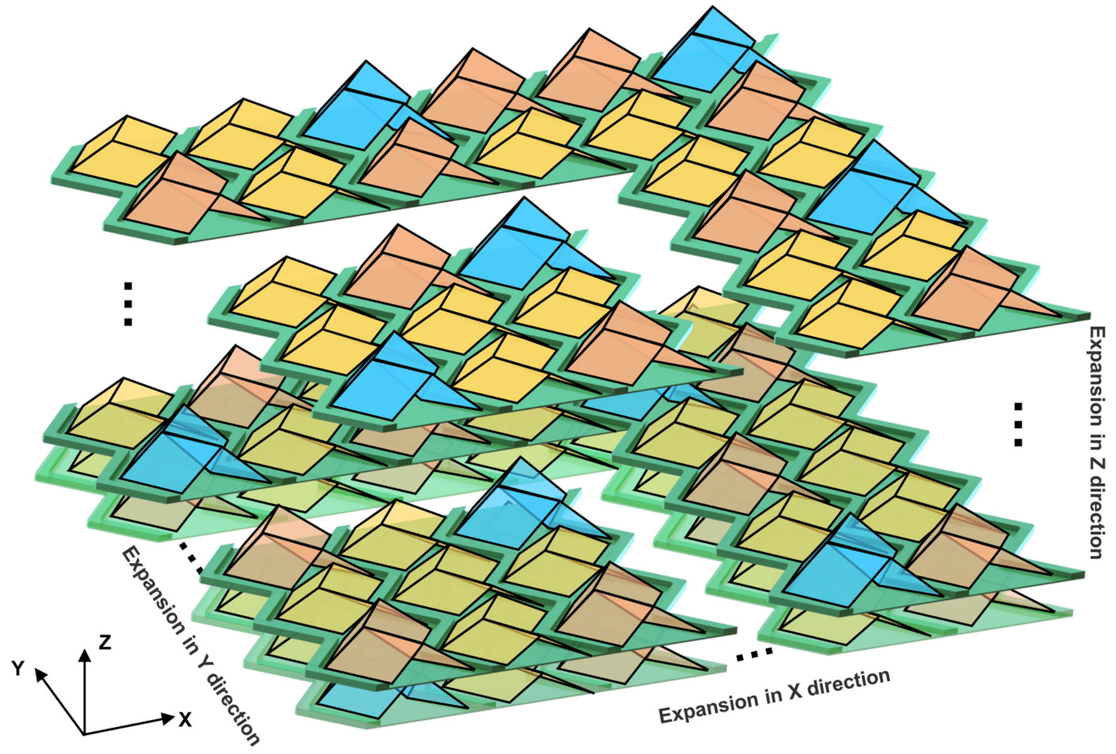

Supplementary Fig. 25. The expansion of the combinatory digital architected material in X, Y, and Z directions. It should be noted that the distribution of units with different configurations in each layer should be as uniform as possible to keep the center of gravity of each layer close to the centroid. If there is only one unit with the highest height configuration on a certain layer, it needs to be placed at the center of the layer to maintain the architected material balance through its two top ridges.

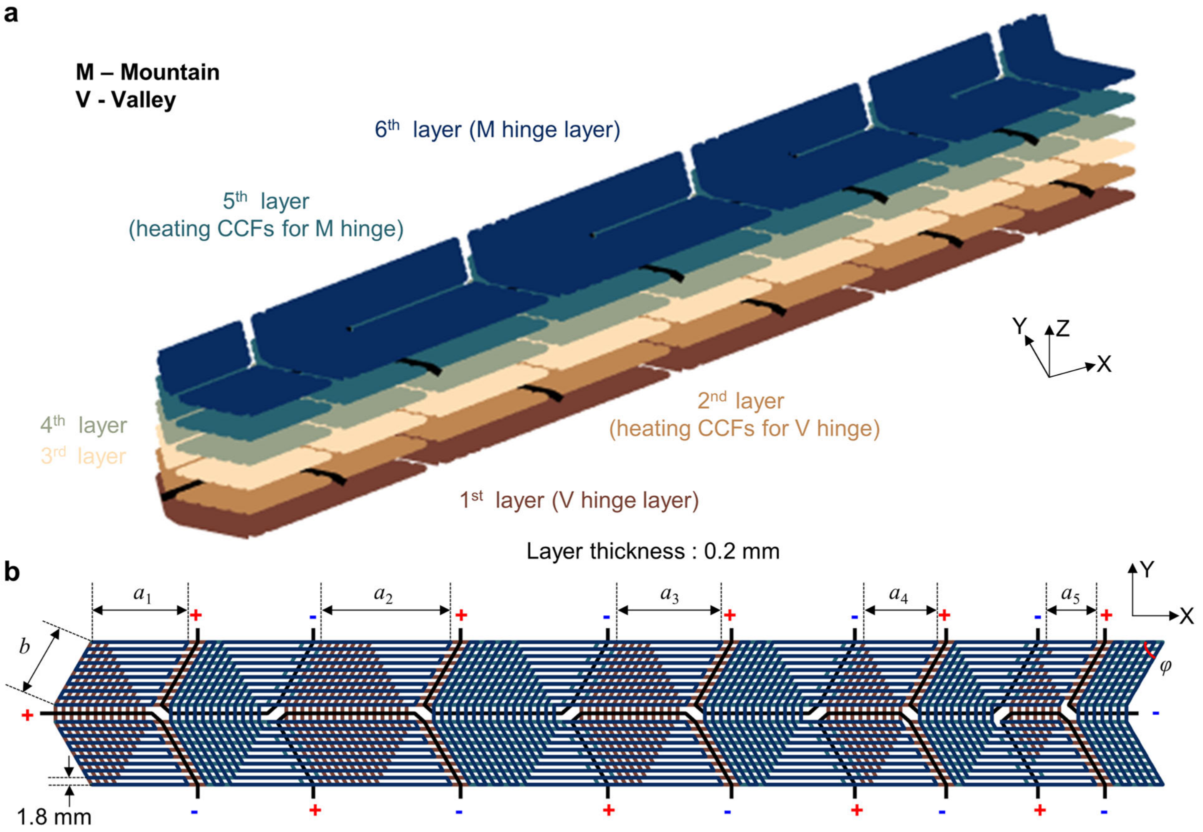

Supplementary Fig. 26. Construction and dimensions of the variable thickness wing based on PCEM. a Oblique view of the fiber path of the variable thickness wing. The first layer and the last layer are respectively the valley and mountain hinge layer, and the second and fifth layers are CCFs layers, heating for the valley and mountain hinge layer, respectively. **b** Vertical view of fiber path and dimension of the variable thickness wing.

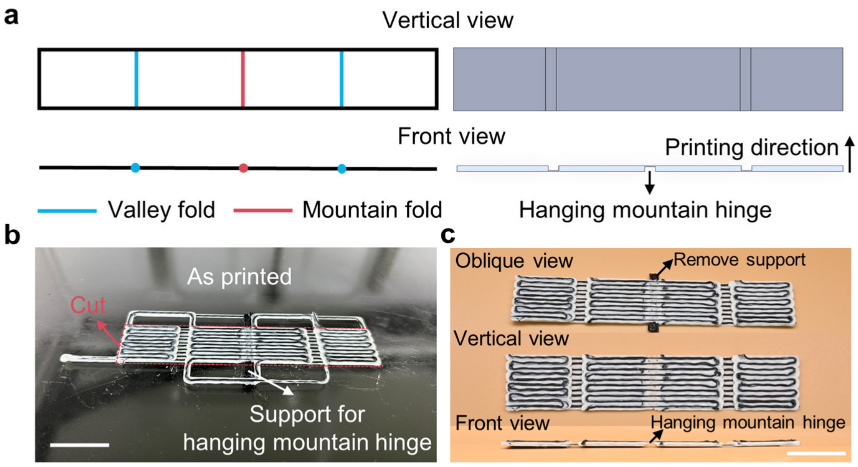

Supplementary Fig. 27. Description of supporting materials for origami composite printing. **a** Vertical and front view of the crease pattern and the computer-aided design of a strip origami structure. **b** Snapshot of the strip origami structure as printed. Scale bar, 20 mm. c Snapshots of the strip origami structure for different views. Scale bar, 20 mm.

i	E_i (MPa)	τ_i^r (s)
1	4.64e-10	4.41e+9
2	2.79e-10	1.10e+9
3	2.47e-10	2.76e+8
4	2.32e-9	6.90e+7
5	1.10e-9	1.72e+7
6	4.08e-10	4.31e+6
7	1.70e-9	1.08e+6
8	4.65e-10	2.69e+5
9	1.31e-9	6.73e+4
10	3.70e-10	1.68e+4
11	2.76e+1	4.21e+3
12	2.85e+2	1.05e+3
13	3.72e+2	2.63e+2
14	6.95e+2	6.58e+1
15	5.13e+2	1.65e+1
16	3.19e+3	4.15e0
17	3.44e-10	1.14e0
18	8.28e+3	6.96e-1
19	3.60e+2	2.14e-4
20	5.67e+2	7.55e-6

Supplementary Table 1. Parameters for the multi-branch model.

Structure	a (mm)	φ (°)	b (mm)	L (mm)	ξ (°)	t (mm)
Upper panel	22.4	60.0	19.6	-	-	1.4
Bottom panel for LS1	-	-	19.6	48.4	31.4	1.4
Bottom panel for LS2	-	-	19.6	48.3	45.0	1.4
Bottom panel for LS3	-	-	19.6	55.3	54.7	1.4

Supplementary Table 2. The geometry design parameters of the Miura-origami unit structure (upper panel) and limit bottom panel structures for different recovery shapes.

Structure	a (mm)	φ (°)	b (mm)	L (mm)	ξ (°)	t (mm)	I (A)	t (s)
Upper panel for ω = 50°	22.2	63.0	19.6	-	-	1.4	0.26	25
Upper panel for ω = 45°	22.4	60.0	19.6	-	-	1.4	0.26	30
Upper panel for ω = 35°	17.6	54.6	19.6	-	-	1.4	0.26	35
Bottom panel	-	-	19.6	48.3	45.0	1.4	-	-

Supplementary Table 3. The geometry design and activation parameters for Miura-origami units with different deployable angles and the geometry design for the limit bottom panel structure.

Structure	a_1 (mm)	a_2 (mm)	a_3 (mm)	a_4 (mm)	a_5 (mm)	b (mm)	t (mm)	φ (°)
I	25.0	35.0	27.0	20.0	13.0	19.6	1.2	60.0
II	7.0	42.0	37.0	22.0	12.0	19.6	1.2	60.0
III	27.0	37.0	27.0	17.0	12.0	19.6	1.2	60.0
IV	22.0	32.0	27.0	22.0	17.0	19.6	1.2	60.0

Supplementary Table 4. The geometry design parameters of four different airfoils variable thickness wing structures.

Supplementary References:

1. Yu K, Ge Q, Qi HJ. Reduced time as a unified parameter determining fixity and free recovery of shape memory polymers. *Nat Commun* **5**, 3066 (2014).

2. Yuan C, Ding Z, Wang TJ, Dunn ML, Qi HJ. Shape forming by thermal expansion mismatch and shape memory locking in polymer/elastomer laminates. *Smart Mater Struct* **26**, 105027 (2017).

3. Williams ML, Landel RF, Ferry JD. The temperature dependence of relaxation mechanisms in amorphous polymers and other glass-forming liquids. *Journal of the American Chemical Society* **77**, 3701-3707 (1955).

4. Schenk M, Guest SD. Geometry of Miura-folded metamaterials. *Proc Natl Acad Sci USA* **110**, 3276-3281 (2013).
