# Supplementary material for: Electrothermally controlled origami fabricated by 4D printing of continuous fiber-reinforced composites
Source: Nat Commun. 2024 Mar 14;15:2322. doi: 10.1038/s41467-024-46591-3 (PMC10940589; doi:10.1038/s41467-024-46591-3)
Supplement: Supplementary file 3 — Description of Additional Supplementary Files [file 41467_2024_46591_MOESM3_ESM.pdf]

Description of Additional Supplementary Files

File Name: Supplementary Movie 1

Description: demonstration of the electrical conductivity and heating response of CCFs.

File Name: Supplementary Movie 2

Description: shape recovery processes of the pure-SMP and CCF-SMP hinges.

File Name: Supplementary Movie 3

Description: FEA and experimental shape-shifting behavior of airplane-shaped PCEO structure.

File Name: Supplementary Movie 4

Description: deployment processes of different configurations of PCEO strip structure.

File Name: Supplementary Movie 5

Description: deployment process of the reconfigurable robot gripper.

File Name: Supplementary Movie 6

Description: FEA and experimental deployment processes of different configurations of PCEM unit.

File Name: Supplementary Movie 7

Description: the deployment, assembly, and compression process of the combinatory digital mechanical Miura-origami metamaterial.
